# Decreased circUBAP2 Expression Is Associated with Preeclampsia by Limiting Trophoblast Cell Proliferation and Migration

**DOI:** 10.1007/s43032-020-00450-w

**Published:** 2021-01-27

**Authors:** Tingting Qi, Di Zhang, Xuting Shi, Minhui Li, Hongbin Xu

**Affiliations:** 1 Obstetrics and Gynecology Department, Huai’an Maternal and Child Health Hospital, Huai’an, China; 2 Dalian Medical University, Dalian, China; 3 Obstetrics and Gynecology Department, The Affiliated Changzhou No. 2 People’s Hospital of Nanjing Medical University, Changzhou, China

**Keywords:** Preeclampsia, circUBAP2, miR-1244, FOXM1, Placenta

## Abstract

Preeclampsia (PE) is a common obstetric disease and a major cause of maternal, newborn, and fetal death. This condition is a multisystem disorder characterized by hypertension, proteinuria, and involvement of the kidney, liver, and nervous system. It is generally believed that the placenta is the main cause of PE. circRNAs are a special class of noncoding RNAs that can form covalently closed continuous ring structures with tissue-specific conservation, and they have been reported to play a wide range of regulatory functions in various diseases, including PE. In this study, we reported a novel circUBAP2 (hsa_circ_0003496) and found that it was downregulated in placental tissues from patients with PE compared to healthy controls. After knocking down circUBAP2 in trophoblast cells, we found that cell proliferation and migration were significantly suppressed. In addition, preliminary mechanistic studies showed that circUBAP2 can sponge miR-1244, and FOXM1 was identified as a target gene for miR-1244. Cotransfection of si-circUBAP2 and a miR-1244 inhibitor partially reversed the suppressive effect induced by circUBAP2 depletion on proliferation and migration. In conclusion, the circUBAP2/miR-1244/FOXM1 axis might be a promising molecular marker for the diagnosis and treatment of PE.

## Introduction

Preeclampsia (PE) is a special disease that occurs during pregnancy and is a risk factor and significantly affects indicators related to maternal and child health [1], thus seriously threatening the life and health of pregnant women and fetuses. This condition is characterized by high blood pressure, proteinuria, and edema after 20 weeks of pregnancy. With increasing gestational age, the symptoms of the patients may gradually increase and may develop into severe PE and eclampsia. Research reports show that the pathogenesis of PE is multifactorial, and its pathogenesis is a complex process involving genetic, immune, or environmental factors [2]. Increasing attention has been given to the role of trophoblast invasion, immune regulation, and vascular endothelial damage in the pathogenesis of PE.

Recently, strong evidence suggests a potential link among epigenetics, ncRNAs (noncoding RNAs), and pregnancy complications. Many studies have identified important aspects of epigenetics, immunology, and cardiovascular and vascular remodeling in PE [3]. Epigenetics regulates gene expression in trophoblast development and function, which sheds new light on placental development and pathological pregnancies [4]. As new ncRNAs, circular RNAs (circRNAs) are covalently closed, endogenous biomolecules in eukaryotes with tissue-specific and cell-specific expression patterns, and many circRNAs exert important biological functions by acting as microRNA or protein inhibitors (sponges) [5]. In the past, circRNAs were originally regarded as incorrect splicing byproducts of conserved molecules, which did not receive much attention. In recent years, with the development of bioinformatics and high-throughput sequencing technology, many circRNAs have been identified. To date, multiple studies have shown that circRNAs play an important regulatory role in tumors, nervous system diseases, diabetes and metabolic diseases, cardiovascular diseases, etc. [6–9], which indicates their potential functions as a new class of molecular markers and therapeutic targets. Accordingly, circRNAs have been suggested as potential biomarkers for different diseases. Recently, researchers have shown interest in the effects of circRNAs in PE and assessed the mechanisms by which circRNAs are involved in PE [10]. Although the current evidence is limited, possible mechanisms underlying circRNA dysregulation in the etiology of PE are being explored [11]. Qian et al. reported the difference in circRNA expression profiles in the placenta between patients with PE and healthy pregnant women, and the results suggested that circRNAs may play a role in the pathogenesis and development of PE [12–14]. Since then, circTRNC18 and circPAPPA have been reported to regulate trophoblast dysfunction in PE via the ceRNA pathway [15, 16].

In this study, we focused on a new circRNA, circUBAP2 (hsa_circ_0003496), that functions in the trophoblast cell line HTR-8/SVneo and choriocarcinoma cell line JEG-3. circRNAs originate from the UBAP2 (ubiquitin-associated protein 2) gene, which includes hsa_circ_0003496 and has been reported to be upregulated in a variety of tumors [17–19]. However, it has not been reported in pregnancy-related diseases. We found that the expression of circUBAP2 decreased significantly in the placenta of individuals with PE, and inhibition of trophoblast proliferation and migration was significant after interference with the expression of circUBAP2.

## Results

### 1. circUBAP2 Was Downregulated in Placental Tissues from Patients with PE

**Fig. 1:**
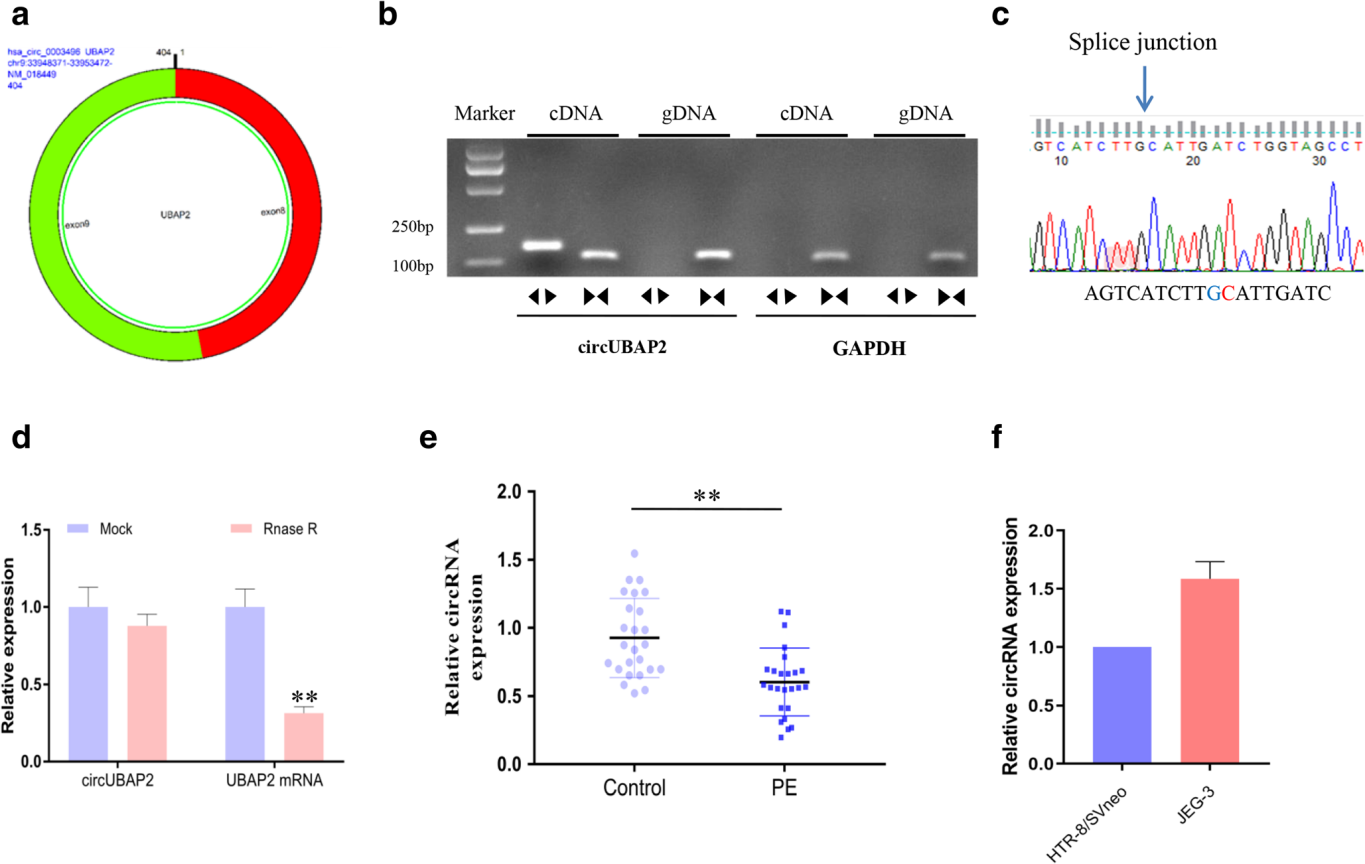
circRNA circUBAP2 was downregulated in the placental tissues of patients with PE. **a** Genomic scheme of circUBAP2. **b** Divergent primers were used to detect circular RNAs in cDNA but not gDNA, and GAPDH was used as a control. **c** Sanger sequence of the junction site of circUBAP2. **d** qPCR analysis of GAPDH and circUBAP2 after RNase R treatment. **e** The expression of circUBAP2 in placental samples of patients with PE and healthy controls was analyzed by qPCR. **f** The relative expression of circUBAP2 in the trophoblast cell line HTR-8/SVneo and trophoblastic tumor cell line JEG-3 was measured by qPCR. Data are expressed as the mean ± SD, unpaired *t* test, **p < 0.01

CircUBAP2 is generated from exons 8 and 9 of the *UBAP2*, which is located on chr9: 33948371-33953472 (Fig. 1a). Divergent and convergent primers were designed to detect circUBAP2 expression, and we found divergent primers that detected circular RNAs in cDNA but not gDNA (Fig. 1b). Then, the PCR products were analyzed by Sanger sequencing, and the junction site of circUBAP2 was confirmed (Fig. 1c). To confirm the circular characteristics of circUBAP2, we treated total RNA with or without RNase R and found that compared to the linear GAPDH and UBAP2 mRNAs, circUBAP2 was obviously resistant to RNase R (Fig. 1d). Then, we found that the expression of circUBAP2 was significantly decreased in the placental tissues from the PE group compared with the adjacent normal controls (Fig. 1e). We also detected the relative expression of circUBAP2 in the trophoblast cell line HTR-8/SVneo and trophoblastic tumor cell line JEG-3 (Fig. 1f).

### 2. Inhibition of circUBAP2 Suppressed Trophoblast Cell Proliferation

**Fig. 2:**
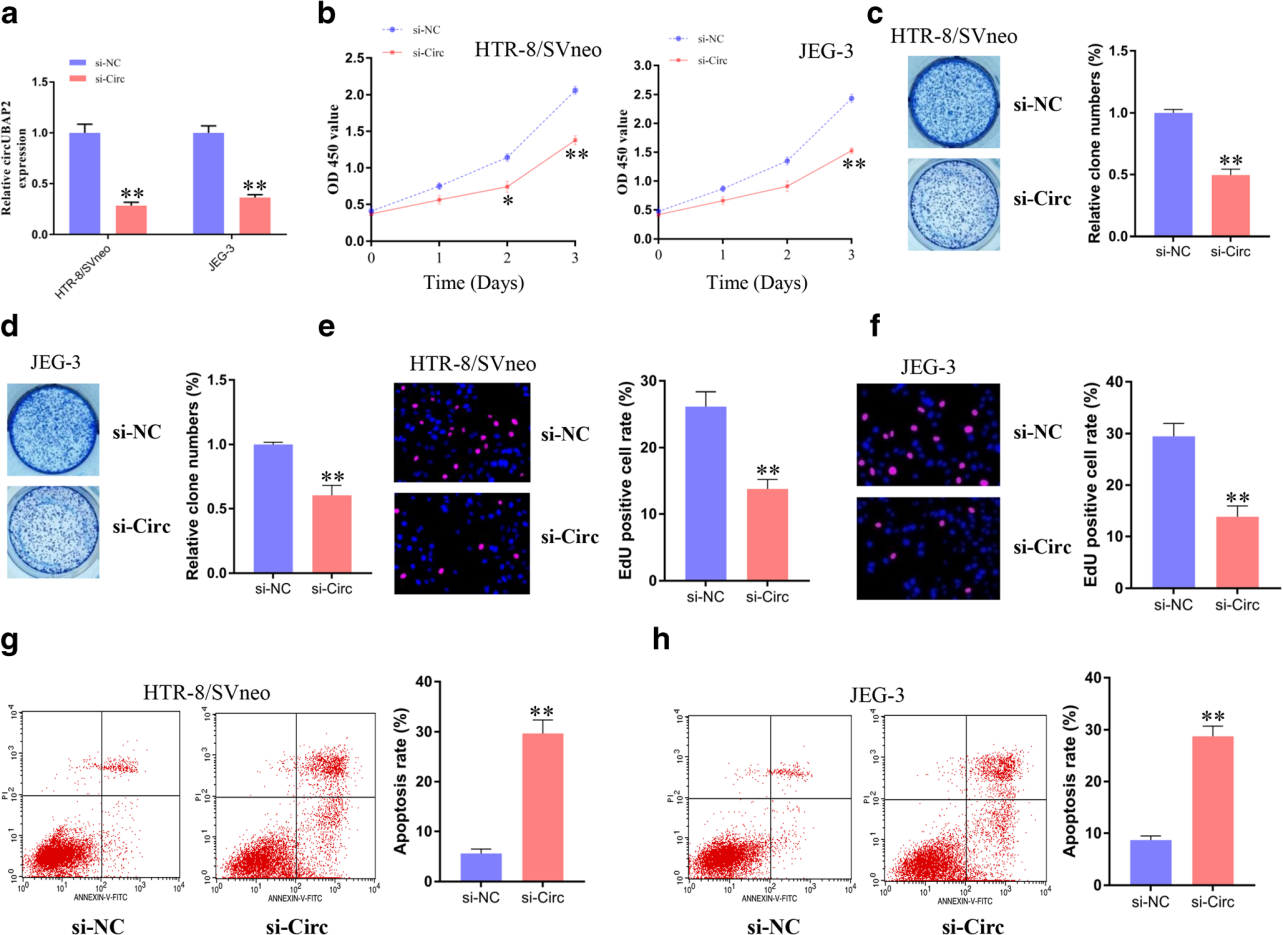
Inhibition of circUBAP2 suppresses trophoblast cell proliferation. **a** The interference efficiency was examined by qPCR in HTR-8/SVneo and JEG-3 cells after transfection with siRNA (si-circ) targeting circUBAP2. **b** The viability of HTR-8/SVneo and JEG-3 cells was measured by CCK-8 assays after transfection with si-circ and si-NC for 24, 48, and 72 h. **c**, **d** Colony formation of HTR-8/SVneo (**c**) and JEG-3 (**d**) cells transfected with siRNAs. **e**, **f** EdU assay to determine the cell proliferation rate of HTR-8/SVneo (**e**) and JEG-3 (**f**) cells transfected with siRNAs. **g**, **h** Cell apoptosis rates were detected using flow cytometry. Data are expressed as the mean ± s.d., unpaired t-test, **p < 0.01

To investigate the potential function of circUBAP2, we designed and synthesized specific small interfering RNAs. The interference efficiency was examined by qPCR, and we found that the expression of circUBAP2 was significantly decreased in both the HTR-8/SVneo and JEG-3 cells after transfection with siRNA (si-circ) targeting circUBAP2 (Fig. 2a). Introduction of si-circ significantly suppressed HTR-8/SVneo and JEG-3 cell proliferation after 72 h (Fig. 2b). The EdU results showed that knockdown of circUBAP2 significantly inhibited cell variability on day 3 (Fig. 2e, f), which was consistent with the results from the CCK-8 assay. Colony formation was also disrupted after circUBAP2 knockdown (Fig. 2c, d). In addition, the cell apoptosis rate was significantly increased in the HTR-8/SVneo cells (Fig. 2g) and JEG-3 (Fig. 2h) cells after transfection with si-circ compared with si-NC.

### 3. Knockdown of circUBAP2 Suppressed Trophoblast Cell Migration

**Fig. 3:**
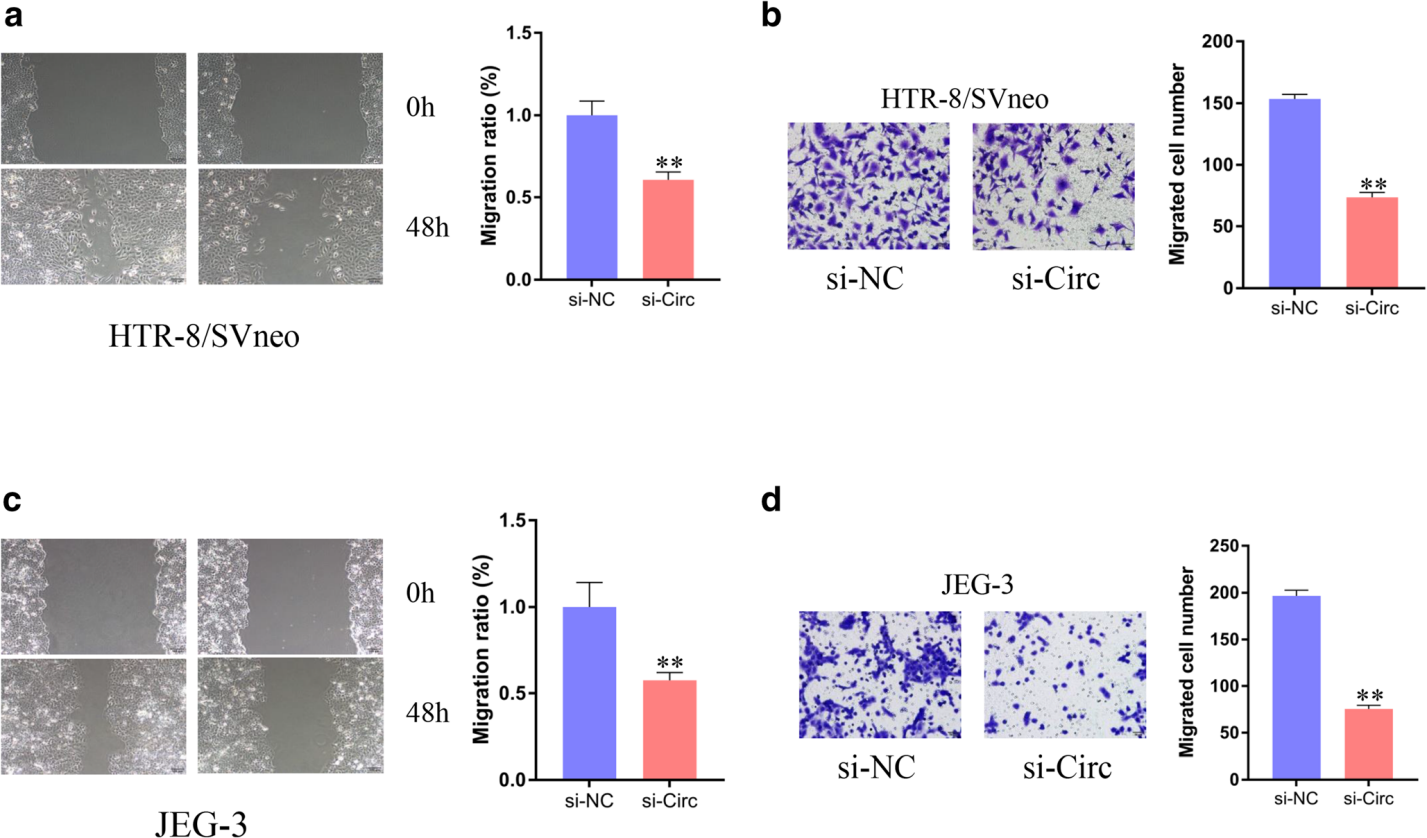
Inhibition of circUBAP2 suppresses trophoblast cell migration. **a**, **c** Cell motility was examined in cells transfected with si-circ or si-NC by wound healing assays. **b**, **d** Transwell migration assays were performed in cells transfected with si-circ or si-NC. Data are expressed as the mean ± SD, unpaired *t* test, **p < 0.01

The effect of si-circ on trophoblast cell migration was determined by wound healing and Transwell assays. Compared with that of the si-NC group, the migrated distance was significantly shorter in both HTR-8/SVneo (Fig. 3a) and JEG-3 (Fig. 3c) cells, suggesting that inhibition of si-circ can block cell migration in vitro. Moreover, the results of the Transwell migration assays showed that downregulation of circUBAP2 significantly inhibited cell migration (Fig. 3b, d), which was consistent with the wound healing assay results.

### 4. circUBAP2 Directly Bound to miR-1244 to Regulate FOXM1 Expression

**Fig. 4:**
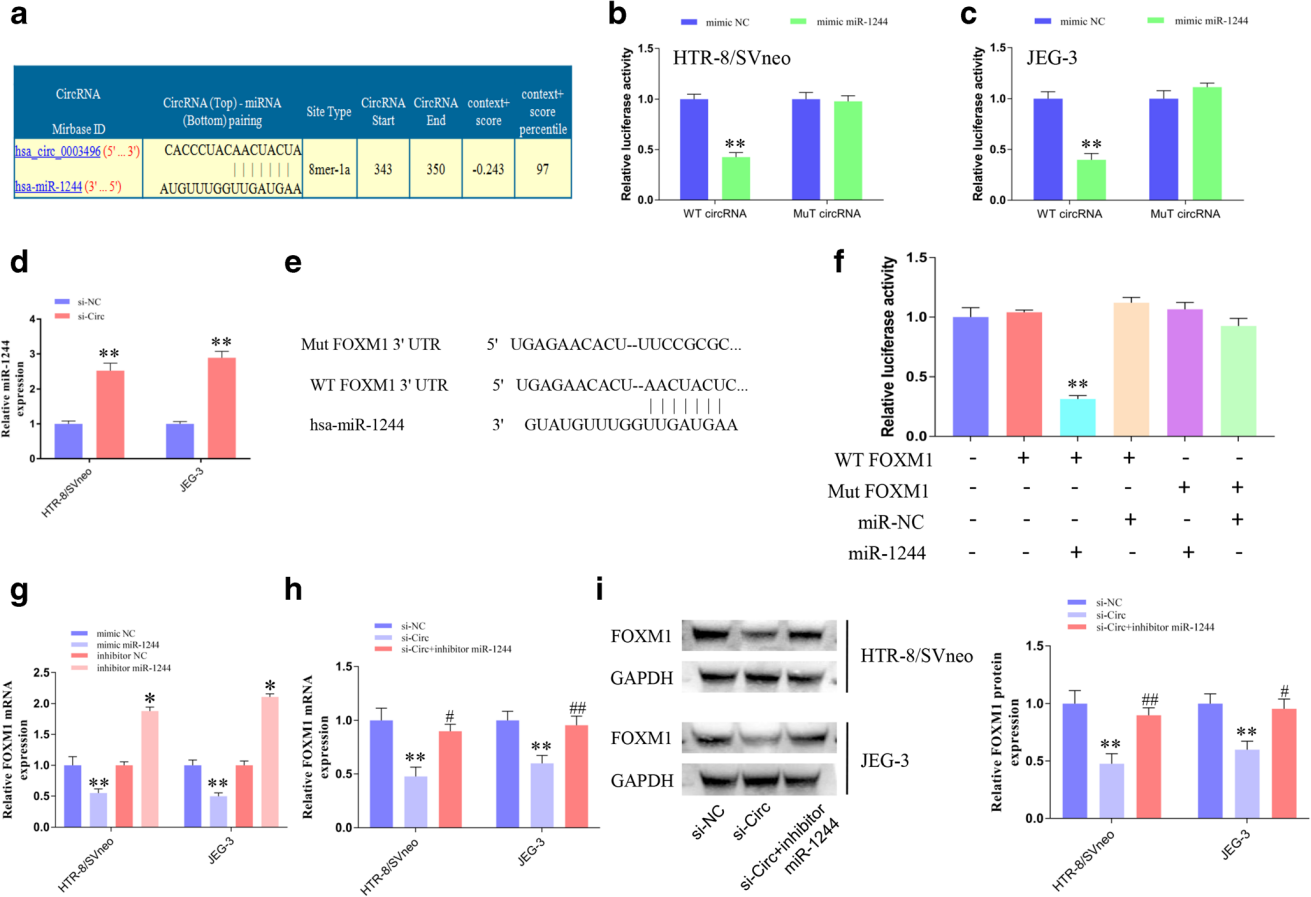
circUBAP2 directly binds to miR-1244 to regulate FOXM1 expression. **a** Potential binding sites of miR-1244 on circUBAP2. **b**, **c** Luciferase reporter assay of wild-type/mutant circUBAP2 with miR-1244/miR-NC in HTR-8/SVneo (**b**) and JEG-3 (**c**) cells. **d** The relative expression of miR-1244 in trophoblast cells after transfection with si-circ or si-NC. **e** The predicted binding sites between FOXM1 and miR-1244. **f** The relative luciferase reporter activity of miR-1244 mimics/NC cotransfected with pmirGLO-WT/Mut-circUBAP2 in HTR-8/SVneo cells. **g** The relative expression of FOXM1 mRNA in trophoblast cells after transfection with miR-1244 mimic/NC or inhibitor/NC was measured by qPCR. **h**, **i** The relative expression of FOXM1 mRNA (**h**) and protein (**i**) in trophoblast cells after transfection with si-circ alone or cotransfection with miR-1244 inhibitor was measured by qPCR or western blots, respectively. Data are expressed as the mean ± SD, unpaired *t* test, *, compared with the si-NC group; #, compared with the si-circ group. *, #, *p* < 0.05, **, ##, *p* < 0.01

**Fig. 5:**
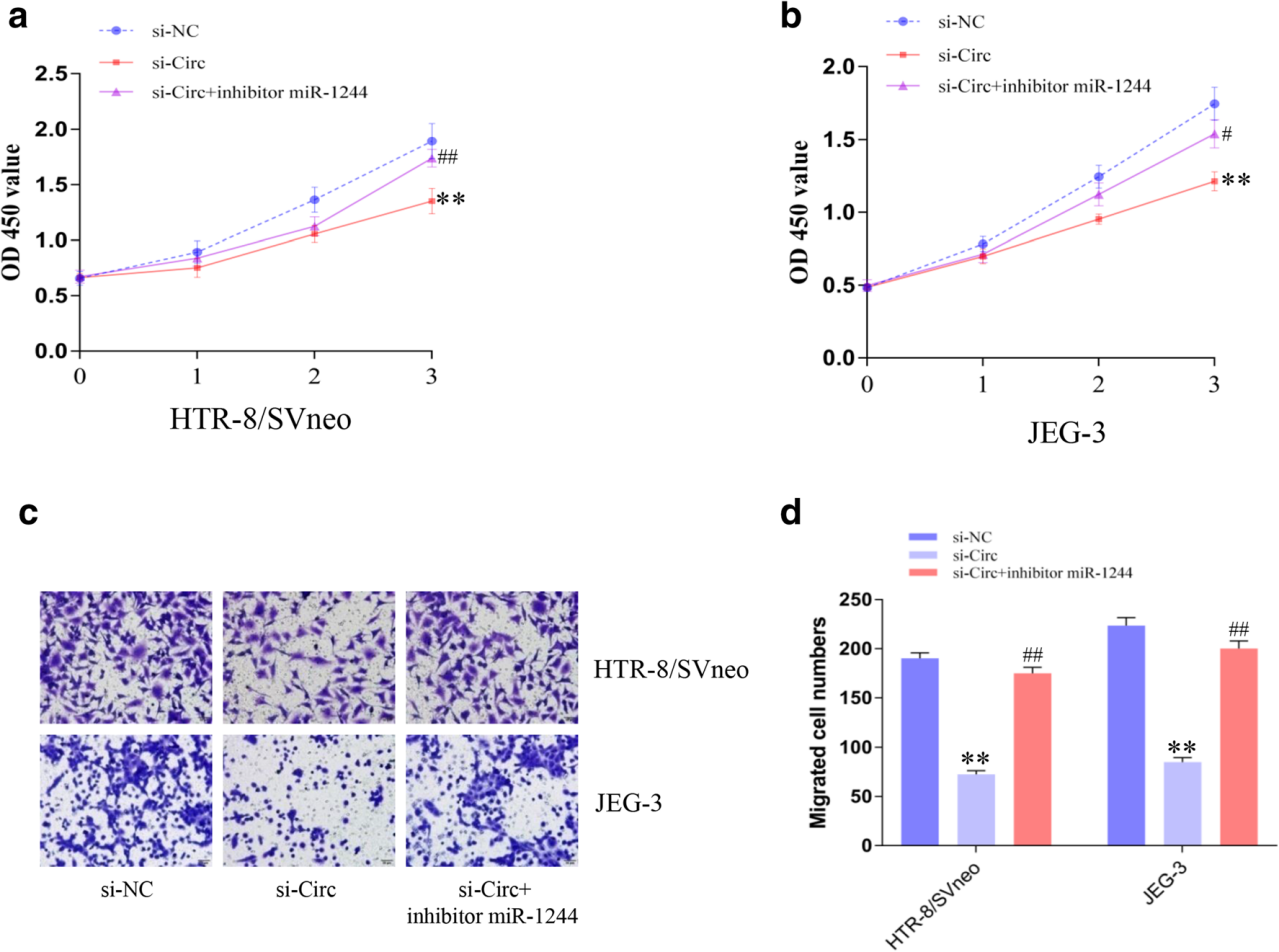
circUBAP2 contributes to cell proliferation and migration by regulating miR-1244/FOXM1. **a**, **b** CCK-8 assay was performed to measure cell viability after transfection with si-NC, si-circ, or si-circ+inhibitor miR-1244 in HTR-8/SVneo (**a**) or JEG-3 (**b**) cells. **c**, **d** Cell migration was assessed by Transwell assays. Data are expressed as the mean ± SD, unpaired *t* test, *, compared with the si-NC group; #, compared with the si-circ group. #, *p* < 0.05, **, ##, *p* < 0.01

Considering that circRNAs could bind to certain miRNAs and therefore regulate downstream genes, through bioinformatics prediction, we found that circUBAP2 has binding sites for the miR-1244 seed region (Fig. 4a). Then, luciferase reporter assays showed that cotransfection with circUBAP2 with WT binding sites for miR-1244 and miR-1244 mimic could significantly reduce the luciferase activities (Fig. 4b, c). qPCR results further confirmed that circUBAP2 knockdown could increase the expression of miR-1244 (Fig. 4d). The downstream targets of miR-1244 were also predicted by TargetScan (http://www.targetscan.org), and the binding sites are shown in Fig. 4e. Then, luciferase reporters containing WT/Mut putative binding sites of FOXM1 transcripts were constructed, and the results showed that the luciferase activities of the FOXM1 wild-type reporter were significantly reduced when cotransfected with the miR-1244 mimic compared with the control reporter or mutated luciferase reporter (Fig. 4f). Moreover, we found that the FOXM1 mRNA expression was downregulated after transfection with the miR-1244 mimic but was elevated after transfection with the miR-1244 inhibitor (Fig. 5g). In addition, circUBAP2 knockdown downregulated both the mRNA (Fig. 5h) and protein expression of FOXM1 (Fig. 5i), but this depletion could be partly rescued by cotransfection with the miR-1244 inhibitor.

### 5. circUBAP2 Contributed to Cell Proliferation and Migration by Regulating FOXM1

Given that miR-1244/FOXM1 expression could be regulated by circUBAP2, we conducted rescue experiments on cell proliferation and migration. CCK-8 assays demonstrated that cotransfection with si-circ and miR-1244 inhibitor could partly rescue the suppressive effect caused by circUBAP2 depletion in trophoblast cells. In addition, cotransfection with si-circ and the miR-1244 inhibitor could partly rescue the inhibition of migration caused by circUBAP2 depletion.

## Discussion

A successful pregnancy depends on the proper invasion of trophoblast cells into decidual tissue to promote embryo implantation and the reconstruction of extracellular matrix and blood vessels to maintain normal physiological functions of the placenta [20]. Abnormal invasion of trophoblast cells may result in too deep or too shallow penetration, which prevents remodeling of the uterine spiral artery, causes anoxia and ischemia of the placenta, and results in damage to endothelial cells of the whole blood vessels of the mother; these changes cause various adverse pregnancy outcomes and pregnancy-related diseases, such as fetal growth restriction, embryo discontinuation, placental abruption, and PE [21, 22]. Normal proliferation and differentiation of trophoblasts is a prerequisite for placental formation [23]. There are multiple factors, mechanisms, and pathways involved in PE, but the specific pathogenesis is still unclear. circRNAs are newly discovered endogenous noncoding RNAs that can regulate genes at the post-transcriptional level [24]. Unlike known linear RNAs, circRNAs can form covalently closed continuous rings and are resistant to RNase R. An increasing number of studies have shown that circRNAs are involved in the development of various diseases [25]. It was found that circRNAs can play a regulatory role as microRNA sponges, RNA-binding protein chelators, and nuclear transcription regulators; thus, circRNAs may be an ideal biomarker for the diagnosis of disease [10]. However, little is known about the role of circRNAs in preeclampsia.

In recent years, some researchers have identified the differentially expressed circRNAs of PE and explored their potential functions [10, 11]. In this study, we found that the expression of circUBAP2 decreased significantly in placentas from the PE group. Subsequently, functional studies indicated that inhibition of circUBAP2 expression suppressed cell proliferation and migration in HTR-8/SVneo and JEG-3 trophoblast cells. Given that circRNAs function as miRNA molecular sponges, through bioinformatics prediction and luciferase reporter analysis, we found that cotransfection with WT circUBAP2 and miR-1244 mimic could significantly reduce luciferase activities, which indicated that they could be targeted directly. circUBAP2 knockdown dysregulated the expression level of miR-1244. miR-1244 was reported to act as a tumor suppressor in osteosarcoma and non-small cell lung cancer [26, 27]. Currently, there is no report on miR-1244 and PE, and we identified FOXM1 (forkhead box protein M1) as its target gene. Overexpression of miR-1244 could significantly reduce the expression of FOXM1, while inhibition of miR-1244 could improve FOXM1 expression.

FOXM1 is an important member of the *FOX* gene family, which plays a key role in cell proliferation, differentiation, and apoptosis. The abnormal expression of FOXM1 is closely related to the occurrence and development of various human malignant tumors [28, 29]. FOXM1 is a transcription factor necessary for tumor proliferation, and its expression level is often related to the degree of malignant progression of tumors. It was reported that FOXM1 expression was downregulated in the placental tissues of patients with preeclampsia, and FOXM1 silencing significantly decreased cell proliferation and migration [30, 31]. FOXM1 mRNA and protein expression was downregulated after transfection of FOXM1 but was rescued by cotransfection with the miR-1244 inhibitor. Finally, we found that cotransfection of si-circ and the miR-1244 inhibitor could partially reverse the suppressive effect of circUBAP2 knockdown on proliferation and migration. Because the placenta is a highly heterogeneous organ [32], our method of analysis in this study is not comprehensive. The systematic elaboration of the function of this molecule in various cells of the placenta is still needed. Based on the above findings, we found that circUBAP2 downregulation inhibited cell proliferation and migration in trophoblast cell by sponging miR-1244 to modulate FOXM1, which was identified as a regulatory protein in early placentation processes and proposing a role in the pathogenesis of PE by limiting trophoblast function [30].

In conclusion, we demonstrated that the circUBAP2 was downregulated in PE and plays an important role in the regulation of trophoblast function. Also, the endogenous competitive relationships among circUBAP2, miR-1244, and FOXM1 were explored preliminarily, providing new insights into the pathogenic mechanism and potential therapeutic targets of PE.

## Materials and Methods

### Patient Specimens

Human placental tissue specimens were collected from patients who underwent cesarean section at Changzhou No. 2 People’s Hospital between March 2018 and July 2019. According to the ACOG diagnostic standard [33], the patients were divided into a PE (25 cases) group and a matched healthy control group (25 cases). There was no significant difference in maternal age, gestational weeks, and BMI between the two groups. The placental specimen was taken from the center of the maternal body (approximately 1-g size), avoiding blood vessels, necrosis, and calcification foci, and rinsed with normal saline to remove blood stains. Inclusion criteria were as follows: pregnant women with a single birth; consent to cooperate with the study; and signed informed consent. Exclusion criteria were as follows: hypertension and other cardiovascular diseases; diabetes and other metabolic diseases; heart disease, hypertension, and family genetic history; autoimmune diseases; and blood system diseases. Placental tissue samples were snap-frozen and preserved in liquid nitrogen. The study design and protocol were reviewed and approved by the ethics committee of Changzhou No. 2 People’s Hospital (Changzhou, China). All patients signed informed consent prior to surgery.

### Cell Culture and Transfection

HTR-8/SVneo cells were cultured in RPMI-1640 medium (Thermo Fisher Scientific, USA) supplemented with 10% fetal bovine serum (FBS, Thermo Fisher Scientific, USA), 100 U/ml penicillin, and 100 μg/ml streptomycin. JEG-3 cells were maintained in DMEM supplemented with 10% heat-inactivated FBS, 100 U/ml penicillin, and 100 μg/ml streptomycin. All cell lines were maintained at 37 °C in a humidified incubator with 5% CO_2_. Small interfering RNAs (siRNAs) targeting circRNA (Table 1) and miR-1244 mimic/inhibitor were purchased from Sangon Biotech (Shanghai, China) and transfected into cells using Lipofectamine 2000 (Invitrogen, Carlsbad, CA) according to the manufacturer’s instructions.

**Table 1:** The primers and shRNA sequences used in this study

ID	5′ to 3′
circUBAP2	Forward: TACCACCACCCCAAGTACAC
	Reverse: GCTTCTGAGGCTTGACTGTG
GAPDH	Forward: CAAATTCCATGGCACCGTCA
	Reverse: AGCATCGCCCCACTTGATTT
miR-1244	Forward: ACACTCCAGCTGGG AAGTAGTTGGTTTGTATGAG
	Reverse: CTCAACTGGTGTCGTGGAGTCGGCAATTCAGTTGAG AACCATCT
U6	Forward: AAAGCAAATCATCGGACGACC
	Reverse: GTACAACACATTGTTTCCTCGGA
FOXM1	Forward: ACCGCTACTTGACATTGGAC
	Reverse: GGGAGTTCGGTTTTGATGGTC
sh-circUBAP2	Forward: AUAACCUGUACUGUAGCCGTT
	Reverse: AUA ACC UGU ACU GUA GCC GTT

### qPCR

Total RNA in placental tissue or cells was extracted using TRIzol reagent (Invitrogen) according to the manufacturer’s instructions, and reverse transcription was carried out using the PrimeScript RT-PCR Kit (TaKaRa, Japan). For RNase R treatment, total RNA was incubated with or without RNase R (Epicenter, USA). The circRNA and FOXM1 mRNA levels were measured by real-time PCR on an ABI7500 PCR instrument using SYBR premix (TaKaRa, Japan). The primers for real-time PCR are listed in Table 1. The relative expression was normalized against that of GAPDH or U6 and analyzed by the formula 2^−ΔΔCT^.

### Western Blot

After the protein was extracted, the concentration was detected by a BCA kit (Beyotime). After denaturation, the protein was separated on 12% SDS-PAGE gels and then transferred to PVDF membranes (Beyotime). After blocking with 5% nonfat milk, membranes were incubated with the following primary antibodies: anti-FOXM1 (1:1000, Abcam, UK) and anti-GAPDH (1:1000, Abcam). After PBST washes, the membranes were incubated with an HRP-conjugated anti-mouse IgG antibody (1:2000, Beyotime, China) at room temperature for 1 h. Finally, the bands on the membranes were visualized with the ECL plus reagent (Beyotime) and photographed by a Tanon-5200 automatic chemiluminescence imaging analysis system (Tanon, Shanghai, China).

### Cell Growth Detection

Cell viability was detected by Cell Counting Kit-8 (CCK-8, KeyGentec, China) assays. Cells were seeded in 96-well plates at a concentration of 2×10^3^ cells/well. At 0, 24, 48, and 72 h, 10 μl of CCK-8 solution was added to each well, and after reaction for 2 h, the absorbance (450 nm) was measured by a microplate reader. For EdU assay, Trophoblast cells were seeded at 2 × 10^3^ cells/well into 96-well plates and grown to logarithmic phase. Ten microliters of EdU dye (KeyGentec, China) was added to each well to stain the cells followed by Hoechst 33342. Three images were randomly taken under a fluorescence microscope, and the EdU-positive cells were counted.

### Cell Apoptosis Assay

Transfected cells were collected and washed with cold PBS 48 h after transfection and then stained with Annexin V-FICT/PI following the instructions of the Annexin V-FITC/PI cell apoptosis detection kit (KeyGentec, China). The apoptotic cell rate was detected by flow cytometry (FACSCalibur, BD, USA).

### Colony Formation Assay

The cell suspension was diluted, and 500 cells were inoculated in a 6-well cell culture plate containing 2 ml of medium. Then, the culture plate was gently shaken in the direction of “x” to evenly disperse the cells. After 12 days, the culture solution was discarded, the culture was terminated, and the culture was carefully immersed and washed with PBS twice. Then, 5 ml of anhydrous ethanol was added, and the sample was fixed for 15 min. After the fixative was discarded, Giemsa dye solution was added for 10–30 min, and then, the dyeing solution was slowly washed away with flowing water, the sample was air-dried, and photos were taken to calculate the number of clones.

### Cell Migration Assay

The wound healing assay was performed to assess cell migration. Cells were seeded in a 6-well plate, and after transfection, vertical lines were scratched across the center of each well. The cells were incubated with serum-free medium for 48 h. The migration distance was analyzed by ImageJ software. For detection of the cell migratory capacity, cells were placed into 24-well Transwell chambers (BD Sciences, MA, USA) with precoated Matrigel. Then, 600 μl of medium containing 10% FBS was added to the lower compartment. After 24 h of incubation, the nonmigratory cells were cleaned with cotton swabs, and the migrated cells were fixed with 4% paraformaldehyde and stained with 0.5% crystal violet solution. The cells stained in each well were photographed and calculated.

### Dual-Luciferase Reporter Assay

The binding sites for miR-1244 in circUBAP2 and FOXM1 were predicted using Bioinformatics software. The wild-type (WT) or mutant (MuT) binding sites were inserted into the pmirGLO luciferase vector (Promega, Madison, WI). The reporter plasmid and miR-1244 mimics were cotransfected into trophoblast cells using the Lipofectamine 2000 reagent. The cells were harvested at 48 h after transfection, and the luciferase activities were measured with a Dual-Luciferase Reporter Assay System (Promega, USA).

### Statistical Analyses

SPSS 22.0 statistical software was used for statistical analysis, measurement data are expressed as the mean ± standard deviation (SD), and *t* tests or nonparametric tests were used for comparisons between rows and groups. *p* < 0.05 was considered statistically significant.
